# Copy Number Variation and Transposable Elements Feature in Recent, Ongoing Adaptation at the *Cyp6g1* Locus

**DOI:** 10.1371/journal.pgen.1000998

**Published:** 2010-06-24

**Authors:** Joshua M. Schmidt, Robert T. Good, Belinda Appleton, Jayne Sherrard, Greta C. Raymant, Michael R. Bogwitz, Jon Martin, Phillip J. Daborn, Mike E. Goddard, Philip Batterham, Charles Robin

**Affiliations:** 1 Department of Genetics, The University of Melbourne, Parkville, Victoria, Australia; 2 Centre for Environmental Stress and Adaptation Research, The University of Melbourne, Parkville, Victoria, Australia; 3 The Bio21 Institute, The University of Melbourne, Parkville, Victoria, Australia; 4 Department of Agriculture and Food Systems, The University of Melbourne, Parkville, Victoria, Australia; 5 Department of Primary Industries, Bioscience Research Division, Bundoora, Victoria, Australia; University of California Davis, United States of America

## Abstract

The increased transcription of the *Cyp6g1* gene of *Drosophila melanogaster*, and consequent resistance to insecticides such as DDT, is a widely cited example of adaptation mediated by *cis*-regulatory change. A fragment of an *Accord* transposable element inserted upstream of the *Cyp6g1* gene is causally associated with resistance and has spread to high frequencies in populations around the world since the 1940s. Here we report the existence of a natural allelic series at this locus of *D. melanogaster,* involving copy number variation of *Cyp6g1,* and two additional transposable element insertions (a *P* and an *HMS-Beagle*). We provide evidence that this genetic variation underpins phenotypic variation, as the more derived the allele, the greater the level of DDT resistance. Tracking the spatial and temporal patterns of allele frequency changes indicates that the multiple steps of the allelic series are adaptive. Further, a DDT association study shows that the most resistant allele, *Cyp6g1-[BP]*, is greatly enriched in the top 5% of the phenotypic distribution and accounts for ∼16% of the underlying phenotypic variation in resistance to DDT. In contrast, copy number variation for another candidate resistance gene, *Cyp12d1*, is not associated with resistance. Thus the *Cyp6g1* locus is a major contributor to DDT resistance in field populations, and evolution at this locus features multiple adaptive steps occurring in rapid succession.

## Author Summary

The study of insecticide resistance has greatly enriched our understanding of the genetic basis of adaptation, because it represents some of the most intense selection pressures acting on any natural population of eukaryote. Thus it can inform us about the limits of natural selection, both in terms of the number and type of mutations that can arise and also in terms of the rate at which these spread throughout populations. Fifty years ago, studies in *Drosophila melanogaster* indicated that many genes contributed to DDT resistance. Subsequent research into the Hikone-R strain indicated much of the resistance in this particular strain could be attributed to a single gene known as *Cyp6g1*. Here we show that there have been successive DDT resistance mutations occurring at the *Cyp6g1* locus. They include an increase in gene copy number and the insertion of transposable elements into the regulatory regions of the *Cyp6g1 g*ene. These mutations have swept to high frequencies in natural populations since World War II, when insecticides were first used. *D. melanogaster* is not a pest and has not been targeted by insecticides, and yet profound changes are occurring within its genome in response to man-made chemicals in the environment.

## Introduction

The genetic basis of adaptation remains one of the central unresolved issues within evolutionary biology. From a genome-wide perspective, recent studies of *Drosophila* and bacteria suggest that a large proportion of nucleotide fixations in these genomes are adaptive, although their individual effects on fitness are typically small [1]–[5]. From the perspective of an individual adaptive trait, the number of genetic variants that contribute to the trait needs to be quantified, and for each variant, its phenotypic effect estimated, including any pleiotropic fitness costs [6], [7]. Ultimately a complete understanding of the genetics of adaptation requires the synthesis of both perspectives. Currently however there are few examples of adaptive traits in which the genetic basis is understood at a high resolution. In bacteria the best examples come from chemostat experiments [8]. In eukaryotes, the predominant examples come from Quantitative Trait Loci (QTL) studies, which suffer from biases, notably that genes of small effect will generally not be identified. Another bias is that a single Quantitative Trait Locus may result from multiple allelic substitutions and hence QTL studies by themselves could underestimate the number of changes in a bout of adaptive evolution. Here we use insecticide resistance as a model adaptive trait in a eukaryote organism. It offers an opportunity to view an adaptive response to a change in a single environmental component and it occurs on a timescale that allows multiple genetic changes to be observed.

Historically, insecticide resistance has provided a good model for adaptation because a novel selective agent is applied to large natural populations. A key insight provided by many insecticide resistance studies is the frequency of parallel mutation. Often, exactly the same mutations arise independently in a gene or in orthologous genes, as adaptive responses to insecticide selection. For example, at the *Resistance to dieldrin (Rdl)* locus, a single A302S substitution has independently occurred across a diverse array of insect species. In the case of *Tribolium casteneum*, this mutation has occurred multiple times in different geographically defined populations [9], [10]. Similarly, the L1014F mutation in the *para* voltage gate sodium channel, the molecular target of pyrethroids and Dichloro-Diphenyl-Trichloroethane (DDT), has arisen independently in numerous species [11]. Such parallel evolution is not restricted to the target molecules of insecticides, but is also seen in detoxifying enzymes, one example being the G137D change in the *Resistance to organophosphate* (*Rop1*) esterase locus in *Lucilia cuprina* and *Musca domestica* [12]. Parallel evolution is one manifestation of the limits to adaptation, because it suggests that there are a limited number of alternatives. Or more precisely, if there are alternatives, they are harder to reach via mutation, provide less of a benefit, or impose greater fitness costs.

The *Cyp6g1* locus of *Drosophila melanogaster* and *Drosophila simulans* provides another example of parallel evolution. In *D. melanogaster* the insertion of transposable element sequence into the 5′ regulatory region correlates with increased transcription of the *Cyp6g1* gene that encodes a cytochrome P450 enzyme capable of metabolising multiple insecticides, most notably DDT [13], [14]. The 491 bp insertion is derived from the long terminal repeat (LTR) of an *Accord* transposable element (TE), and lies 287 bp from the transcription start site [13]. Transgenic studies have demonstrated that the 491 bp *Accord* insertion can drive expression of a GFP reporter gene in detoxification tissues [15], implicating the insertion as the mutation that causes resistance and thereby providing a robust example of *cis*-regulatory adaptation [16]. Furthermore the *Accord* insertion is not found in flies collected before 1940 but is found at high frequency (32–100%) in 34 contemporary populations around the world, and its presence correlates with resistance [17]. In *D.simulans*, a 4,803 bp *Doc* element, a non-LTR TE, has inserted about 200 bp upstream of the putative transcription start site. This insertion correlates with a two-fold increase in transcription and appears to have recently swept to high frequency in a Californian population. Direct evidence of a phenotypic effect of the *Doc* insertion is equivocal, but the large window of reduced nucleotide variation around *D.simulans Cyp6g1* is consistent with insecticide based selection [18].

DDT resistance in *Drosophila* is an engaging case study of adaptive evolution, partly because the results of genetic mapping studies have reached different conclusions about the basis of resistance. Most early research, including the classic studies of J.F. Crow, indicated that DDT resistance was polygenic with genes contributing to DDT resistance distributed among all three major chromosomes [19]–[22]. In contrast, work by Kikkawa in 1964 showed that the resistance in the Hikone-R strain was due to a single dominant locus, which mapped to 64.5 cM on chromosome II [23]. This was subsequently identified as *Cyp6g1* [24]. Since the identification of *Cyp6g1* as a resistance locus, researchers have identified the molecular mechanism of the upregulation [15], looked for selective sweeps centred at this locus [17], and have contended that other loci also play important roles in DDT resistance [25]. Other than *Cyp6g1*, two additional loci have been implicated in DDT resistance, and both are also cytochrome P450 genes. CYP6A2 has DDTase activity that has been shown to be reliant on three amino acid substitutions that distinguish a lab resistant strain from susceptible strains [26]. The other locus, *Cyp12d1*, is highly inducible by DDT [27]. Its overexpression in flies using transgenic techniques increases DDT resistance [28], and the locus exhibits high frequency copy number variation in natural populations. However *Cyp6g1* is the only locus shown to have alleles that contribute to variation in DDT resistance in field populations of *D. melanogaster*.

We, and others [29], [30], have found copy number variation and extra alleles at the *Cyp6g1* locus of *D. melanogaster*. This casts a new light on the nature of the resistance mutations, the phenotypic contribution to DDT resistance and the adaptive significance of *Cyp6g1* variation. The aims of the research reported here are to: (i) characterise this molecular variation, focussing on additional transposable element insertions in the locus and gene copy number variation, (ii) determine if this molecular variation correlates with phenotypic variation in the form of DDT dosage-mortality relationships, (iii) assess whether the variation is adaptive by analysing the genotypic frequencies of this newly described variation in historical and contemporary populations of *D. melanogaster* and (iv) quantify the contribution that *Cyp6g1* locus variation makes to overall DDT resistance in a natural population.

## Results

### Characterisation of *Cyp6g1* copy number variation (CNV) and TE insertion complexity in the RK146 strain

We sequenced *Cyp6g1* from seven *D. melanogaster* isochromosomal lines (chr.II), which revealed that six lines had double peaks on their sequence chromatograms. Since the isochromosomal lines should only have one allele for genes on the second chromosome we inferred that there must be CNV for *Cyp6g1*. We confirmed this by determining that both alternate states of sequence variants were passed to all individuals in the next generation (data not shown). To determine the details of this CNV we initially characterized the *Cyp6g1* loci in a single isochromosomal line (RK146). *In situ* hybridisation of a *Cyp6g1* probe to polytene chromosomes indicated that the copies of *Cyp6g1* were within the same cytological band (Figure S1). Assuming that these were arrayed in a direct tandem manner, we performed a PCR and identified one breakpoint of the locus duplication, adjacent to the previously identified *Accord* LTR.

To ascertain the other breakpoint associated with this CNV, inverse PCR was performed. Unexpectedly this revealed a ∼7 kb insertion of an *HMS-Beagle* TE within another *Accord* insertion. Notably the size of the *HMS-Beagle* insertion means that it would not have been detected using the *Accord* spanning PCR assays of previous studies, thus the copy lacking *HMS-Beagle* is presumably the locus scored previously [13], [17], [31].

**Figure 1:**
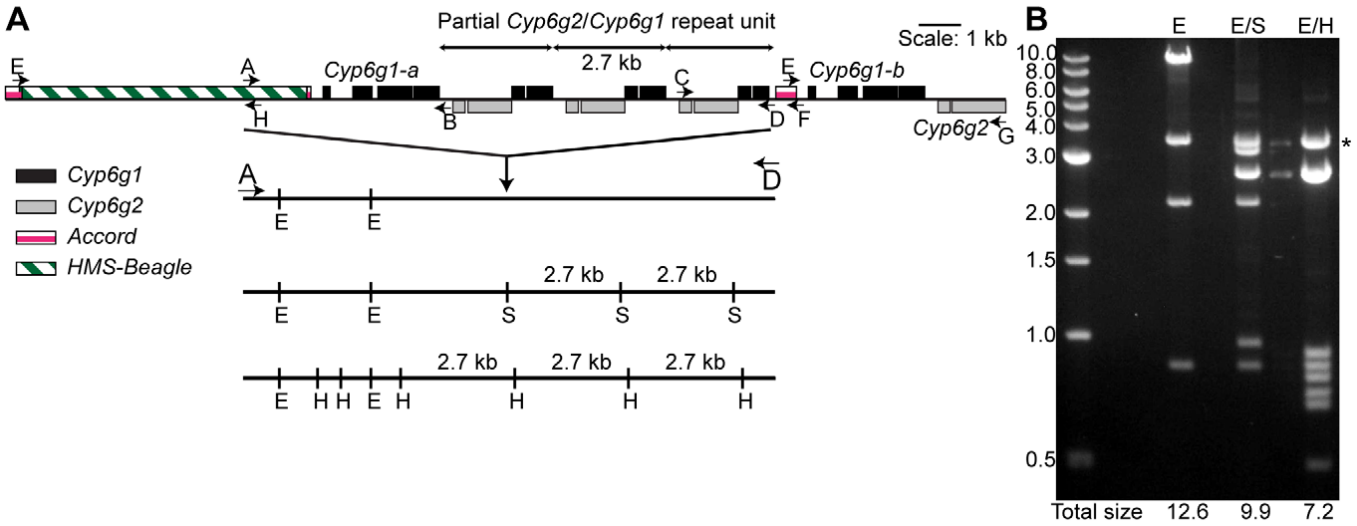
The molecular structure of the *Cyp6g1* locus. (A) The region encompassed by five overlapping PCR primer sets (E-H, A-B, A-D, C-D, E-G) that have been cloned are shown. *Cyp6g1* exons are represented as black boxes above the line, *Cyp6g2* exons as dark grey boxes below the line. The *HMS-Beagle* element and the *Accord* element sequences are represented as an empty box and as light grey boxes above the line respectively. (B) The complex structure of the locus is demonstrated by restriction digests (*Eco*RI; E, *Eco*RI-*Sac*I; E/S and *Eco*RI-*Hind*III; E/H) of a plasmid containing the 12.5 kb A-D PCR product. The restriction sites for each digest are shown as lines drawn to scale with the locus structure. The 3.5 kb EcoRI-EcoR1 plasmid band is seen in all lanes. The sum of the molecular weights of the remaining fragments is shown at the bottom of each lane. The sums differ by intervals of 2.7 kb, which corresponds to the repeat unit represented in the top of the figure. * indicates 3.5 kb vector band. Sequences of E-H, A-B, C-F and E-G are lodged in Genbank with accessions HM214801, HM214799, HM214802 and HM214800 respectively.

To determine the extent of *Cyp6g1* CNV we generated a model of the locus using Southern blots that we tested using PCR. A primer located within the *HMS-Beagle* sequence was used in combination with primers within *Cyp6g1* and the resulting amplicons were cloned and sequenced. The longest amplicon was 12.5 kb and spans the distance between the *HMS-Beagle* element in the first copy (hereafter called *Cyp6g1-a*) and the beginning of the second full copy of *Cyp6g1* (hereafter referred to as *Cyp6g1-b* Figure 1A). A similar approach, using a primer within *Accord* was used to amplify all of *Cyp6g1-b*. In this case the *HMS-Beagle* insertion was used to advantage as it prevented amplification of *Cyp6g1-a*. Table S1 shows the differences between *Cyp6g1-a* and *Cyp6g1-b*, none of which alter the predicted amino acid sequence.

Aside from these two full-length copies of *Cyp6g1* a repeat unit that contains a fusion of partial copies of both *Cyp6g1* and *Cyp6g2* was identified. *Cyp6g1* and *Cyp6g2* are transcribed from opposite strands and are arranged in a convergent orientation. The repeat unit starts at ∼codon 323 of *Cyp6g1* and continues to about codon 73 of *Cyp6g2*. The repeat unit can be seen in Southern blots as a 2.7 kb fragment common to different restriction enzyme digests (Figure S2). It is also demonstrated by the multiple digests of the A-D amplicon clone shown in Figure 1B, where this structure is inferred by the consistent 2.7 kb size difference between different restriction enzyme digests.

While we are confident that the structure shown in Figure 1 occurs in the RK146 strain, it remains a formal possibility that there are *Cyp6g1* associated elements outside this region.

### Strain comparisons reveal an allelic progression

**Figure 2:**
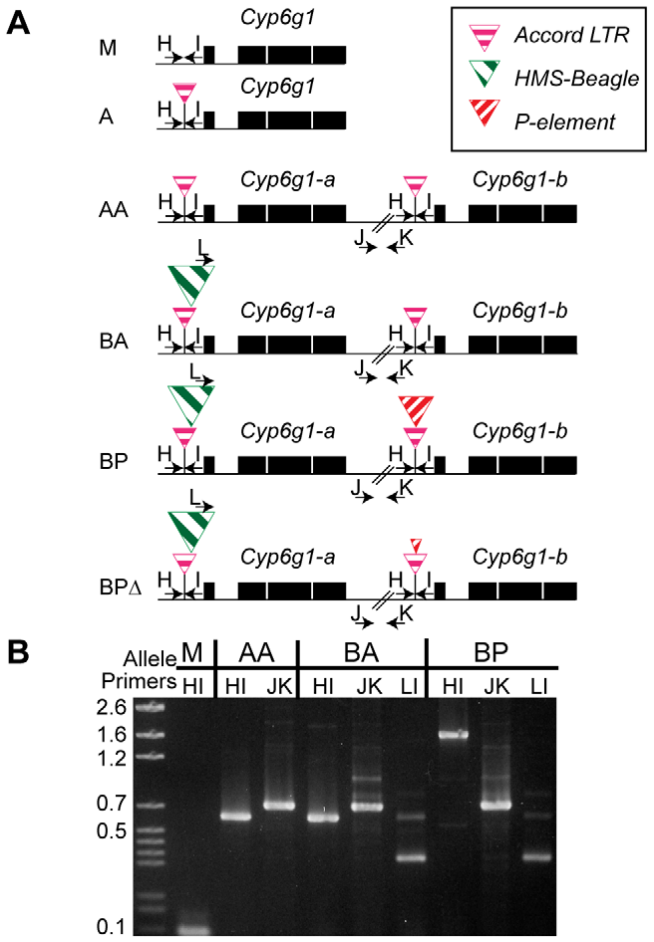
The six alleles of *Cyp6g1*. (A) The alleles are arranged from top to bottom in the order in which they arose. The *M* allele has a single copy of *Cyp6g1* and lacks TE insertions. The *A* allele is single copy with an *Accord* insertion, the *AA* allele has two full length copies each with an *Accord* insertion. The *BA* allele has two full-length copies with the proximal copy containing *HMS-Beagle*. The *BP* allele has two full-length copies with the *a* copy containing *HMS-Beagle* and the *b* copy containing the *P* insertion. All of the lines assayed that have a *P*-element sequence in the *Accord* also have the *HMS-Beagle* insertion and so the *BP* class probably arose from the BA allele. The heterogeneous *BP*Δ class contains various low frequency variants that have scrambled *P* terminal repeats (Figure S3). PCR primers are shown as arrows and are named with a single letter. Note that primer L anneals to the *HMS-Beagle* sequence whereas primers H and I flank the transposable element insertion sites. (B) A gel demonstrating the diagnostic PCRs is shown on the right.

Previously a partial *P* element had been identified nested within the *Accord* element upstream of *Cyp6g1* [17], [31]. We designed PCR assays to score flies for this partial *P* insertion, the *Accord* insertion, the *HMS-Beagle* insertion and the presence of the gene duplication. We could not develop a co-dominant assay for the duplication i.e. we could identify lines carrying the duplication with a PCR bridging a breakpoint but we could not develop an assay that uniquely amplified the single copy allele and not the double copy alleles. Therefore we performed PCR assays on isochromosomal lines, highly inbred lines and F1 crosses to a known genotype (Table S2 for details). These assays revealed even more variation; specifically alleles derived from the partial *P*-element insertion where most of that *P*-element had been removed leaving small scrambled portions of the terminal repeats (Figure S3). Thus we describe six alleles (*M*, *A*, *AA*, *BA*, *BP*, *BP*Δ) defined on the basis of this molecular variation (Figure 2). The facts that the two full length copies both have an *Accord* LTR insertion, some TEs are nested, and that all lines containing the *P*-element insertion also have the *HMS-Beagle* insertion, suggests an order to the molecular events at this locus, which is indicated in Figure 2.

### Geographic and temporal patterns of allelic variation

**Figure 3:**
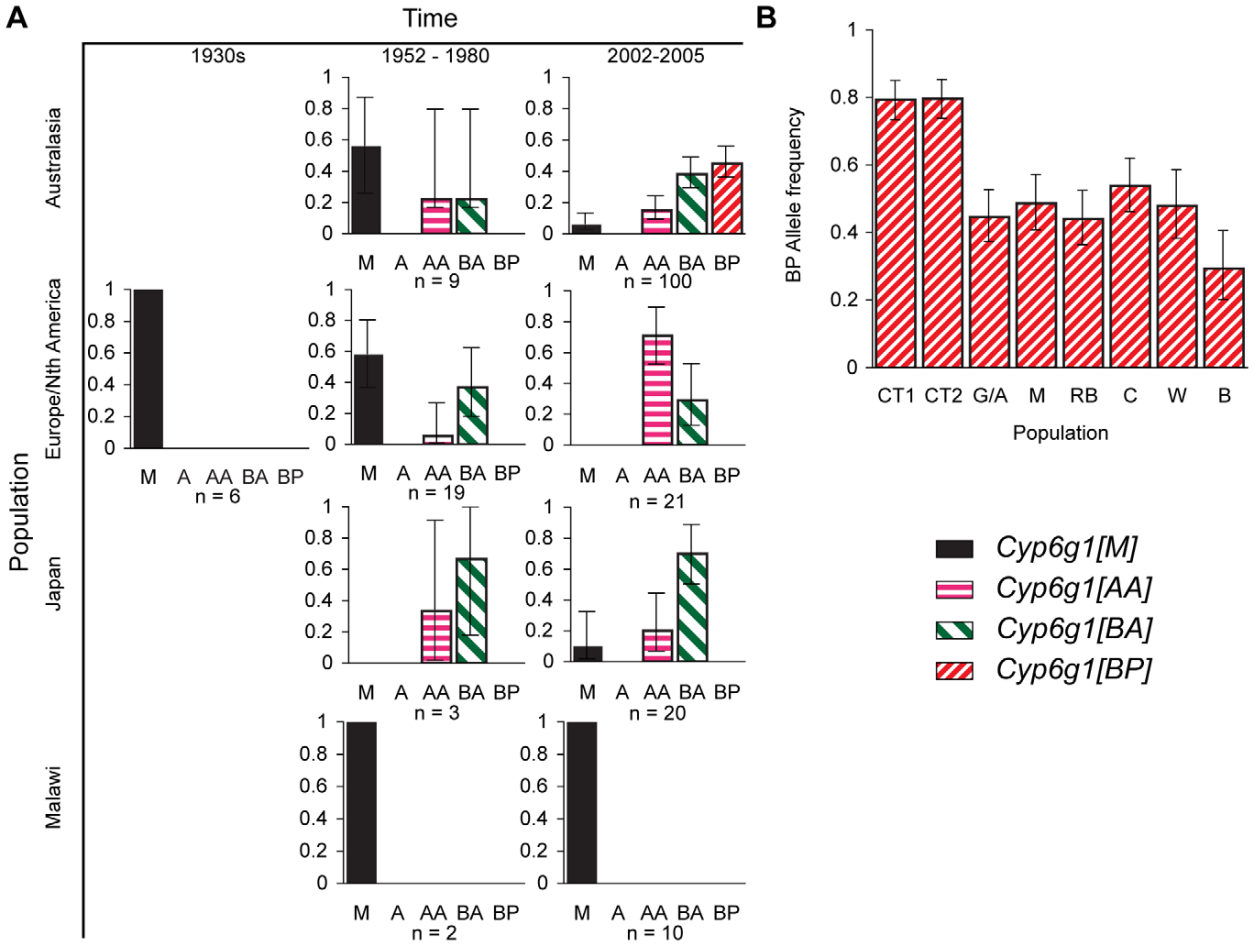
Temporal and geographic changes in *Cyp6g1* allele frequencies. (A) Lines established between the 1930s and the present. Flies were typed using the PCR assays described in Figure 2. (B) The frequency of the BP allele was scored in >40 flies from each of eight locations along the east coast of Australia. The populations are from Cape Tribulation (CT1, CT2), Gladstone/Alstonville (G/A), Maryborough (M), Rainbow Beach (RB), Coffs Harbour (CH), Wollongong (W), Bega (B). Error bars in both (A,B) represent the 95% binomial confidence interval.

We conducted three surveys of *Cyp6g1* alleles in *D. melanogaster* populations: a survey from historical samples, a survey of lines from contemporary global populations, and a survey of wild males collected on the east coast of Australia (Figure 3). These studies included a re-analysis of lines scored for the *Accord* element in earlier studies to determine whether the lines had the duplicated locus or had other variants not originally described [13], [18]. Consistent with previous studies, the ancestral *Cyp6g1-[M]* allele is rare in most contemporary populations except for the population from Malawi, Africa [17], [18]. The historic samples show that the *M* allele was more frequent in the 1950–1980 samples and was the only allele observed among the lines established in the 1930s (Figure 3A). Surprisingly the *Cyp6g1-[A]* allele, which was the presumed state of previous studies, is not observed in three population surveys. Most flies in Europe, Asia, and the USA have *Cyp6g1* duplicated and are of the *Cyp6g1-[AA]* or *Cyp6g1-[BA]* class. A re-analysis of 13 lines collected before 1966 that had been classified as having the *Accord* insertion [13], found that all contained the *Cyp6g1* duplication and 11 also carried the “*HMS-Beagle*” insertion (Table S2). The latter includes Hikone-R, which is derived from flies collected in Japan in 1952 [32], and which was the DDT resistant strain that was initially used to map resistance to the map position where *Cyp6g1* resides [21], [24].

Our survey of 190 alleles from historic and contemporary global populations found the *Cyp6g1-[BP]* allele only in current Australian populations (Figure 3A). The *BP* allele is not seen in any population before 1980 (n = 39), they are not seen in the non-Australian flies surveyed by us in 2002–2005 (n = 51), and Catania *et al* reports them at a frequency of 10/683 in a worldwide sample[17]. However the sample from Australia represented in Figure 3A shows that they are at a frequency of 40/100, which is highly significantly different from the Catania *et al* study (p<0.0001) and our own survey of non-Australian alleles (p = 0). Our third survey, which was of ∼40 males caught in each of eight populations, specifically addressed the frequency of the *BP* allele in Australia. The *BP* allele frequency ranged from 29–80% with the highest being in the most northerly populations (Figure 3B). This level of population differentiation between Australian and other populations is highly unusual in *D. melanogaster* and suggests positive selection has recently driven the *BP* allele to these high frequencies [33]–[35].

We identified two types of *Cyp6g1-[BP*Δ*]* alleles. Both were extremely rare being observed a combined total of three times; all from northern Australia. Because of their rarity we have not considered them further in this manuscript except to record their sequence in Figure S3.

### Allelic variation at *Cyp6g1* determines DDT resistance phenotype

**Figure 4:**
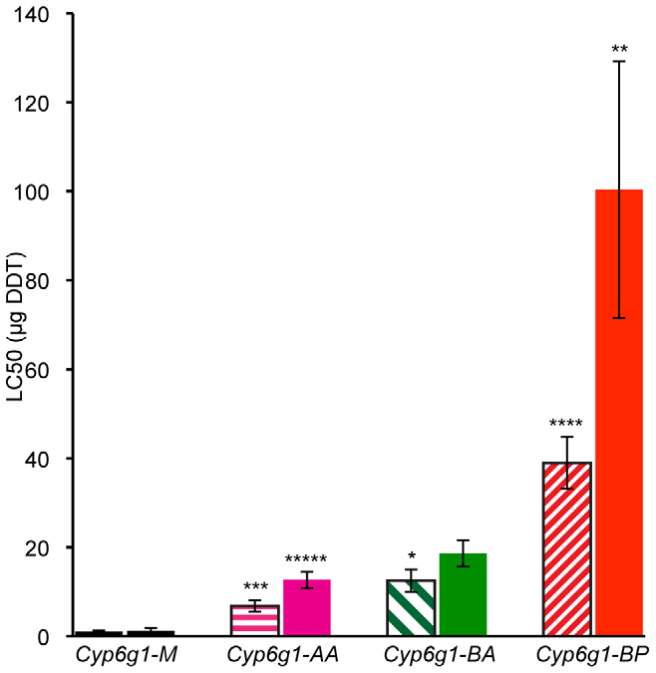
DDT resistance correlates with *Cyp6g1* allelic class. The resistance of lines isochromosomal for their second chromosome are shown grouped on the x-axis by *Cyp6g1* alleles (*M*, *AA*, *BA*, *BP*). The error bars represent the standard errors of the mean LC_50_ of the lines. Significant differences with preceding classes are indicated e.g. *BA* vs. *BP* females (One-tailed t-test: ***** p = 0.0005, **** p = 0.002, *** p = 0.003, ** p =  0.019, * p = 0.044). Shaded bars represent data from males, solid bars from females.

For genetic variation to be adaptive it needs to contribute to phenotypic variance. So we asked whether the molecular variation described above contributes to DDT resistance – the phenotype originally attributed to this locus. To answer this, DDT resistance was calculated by contact assay [24] on four day old adults of 19 isochromosomal lines and 3 inbred lines, most of which were derived from Australian populations (Table S2 and Table S3). Males and females were assayed separately for at least five strains of each genotype: *M*, *AA*, *BA* and *BP*. Figure 4 shows that *M* strain males are on average 7 fold less resistant than *AA* strain males. For females there is 10 fold difference (see Table S3 for more details). Furthermore, with each new *Cyp6g1* allele there is an increase in resistance level. There is about a 50% increase from *AA* to *BA* flies although this is only significant in the case of males. The males of the *BP* strains are on average 40 fold more resistant than the *M* strains and the females are 80 fold more resistant. The fact that the *Cyp6g1* allelic classes strongly correlate with resistance, despite the diverse genetic background of the strains assayed, suggests *Cyp6g1* is a major determining factor of the DDT resistance phenotype in these lines.

### Transcription of *Cyp6g1* genotypes

**Figure 5:**
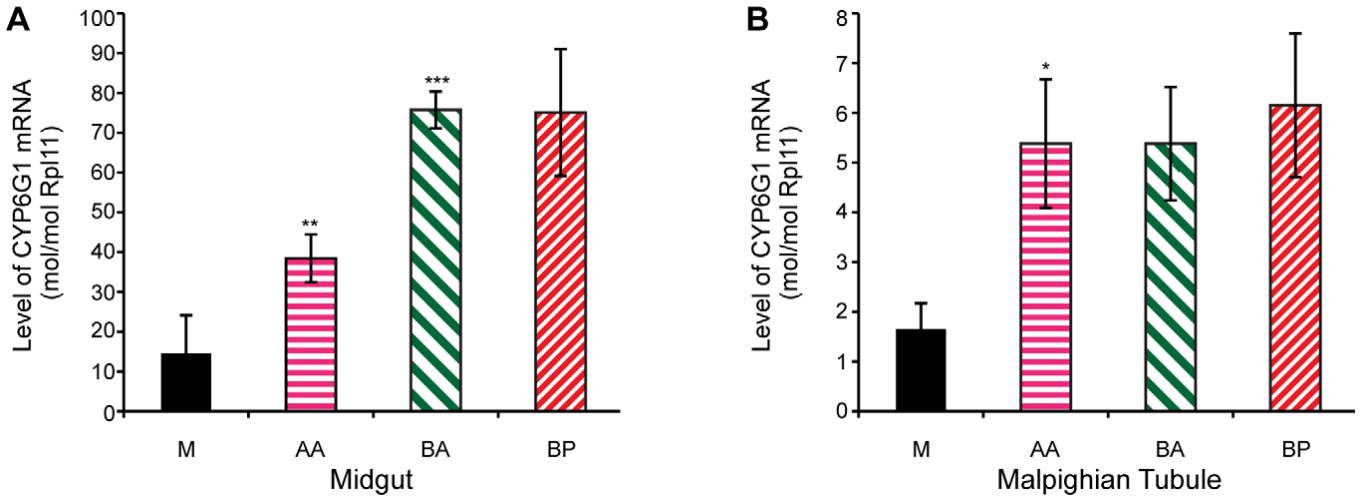
*Cyp6g1* transcription in adult midgut and malphigian tubule. Derived alleles exhibit higher gene expression than *Cyp6g1[M]*. (A) The allelic progression results in increased gene expression in the midgut with *AA* having a 2.6 fold increase (p = 0.036, one tailed t-test) over *M* and *BA* and *BP* both showing a 5 fold increase. There is also a significant 2 fold increase of *BA* over *AA* (p = 0.0006, one tailed t-test). In contrast, only the step between *M* and *AA* results in a significant increase in transcription (p = 0.04, one tailed t-test) in adult tubule (B), and all three derived alleles exhibited ∼3 fold increases in gene expression.

Transgenic studies have previously shown that the *Accord* LTR can act as a tissue specific enhancer of gene expression [15]. To test whether transcription levels correlate with the remaining steps in the allelic series, we analysed *Cyp6g1* expression in 18 of the lines analysed for DDT resistance. The tissue and cell types where DDT detoxification occurs are currently unknown, but we chose to analyse the adult midgut and adult Malpighian tubules because a *UAS*-*Cyp6g1* transgene confers resistance when over-expressed in these tissues using the *Accord* LTR Gal4 driver [28]. Furthermore, RNAi knockdown of *Cyp6g1* in the tubules increases susceptibility of flies to DDT [36]. We found a positive and significant correlation between DDT resistance and transcription levels among the 18 lines assayed for both the midgut and the tubules (Spearmans rank; midgut  = 0.62, p<0.02, tubule  = 0.52, p<0.05). However when the 18 lines are grouped by allelic class, as shown in Figure 5, significant differences in transcription levels are observed for only some of the steps in the allelic progression. In the midgut, the derived alleles clearly exhibit higher gene expression than *Cyp6g1-[M]*; with *AA* having a 2.6 fold increase (P = 0.036, one tailed t-test) and *BA* and *BP* both showing a 5 fold increase. There is also a significant 2-fold increase of *BA* over *AA* (P = 0.0006, one tailed t-test). In tubule, only the step between *M* and *AA* results in a significant increase in transcription (P = 0.04, one tailed t-test; Figure 5B), and all three derived alleles exhibited ∼3 fold increases in gene expression.

### *Cyp6g1* genotype determines resistance in field populations

There is a formal possibility that some other background genetic factor is by chance correlated with *Cyp6g1* allele and that it contributes to the phenotypic trend shown among the lines we analysed for DDT resistance. This is unlikely, partly because most isochromosomal lines were generated in such a way that 100% of the X chromosome material and 75% of the third chromosome material would be derived from the Prl/CyO stock (Table S2). Nevertheless we decided to take a quantitative genetics association study approach to confirm and quantify the role of the most derived high frequency allele, *Cyp6g1-[BP]*, in a field population.

**Figure 6:**
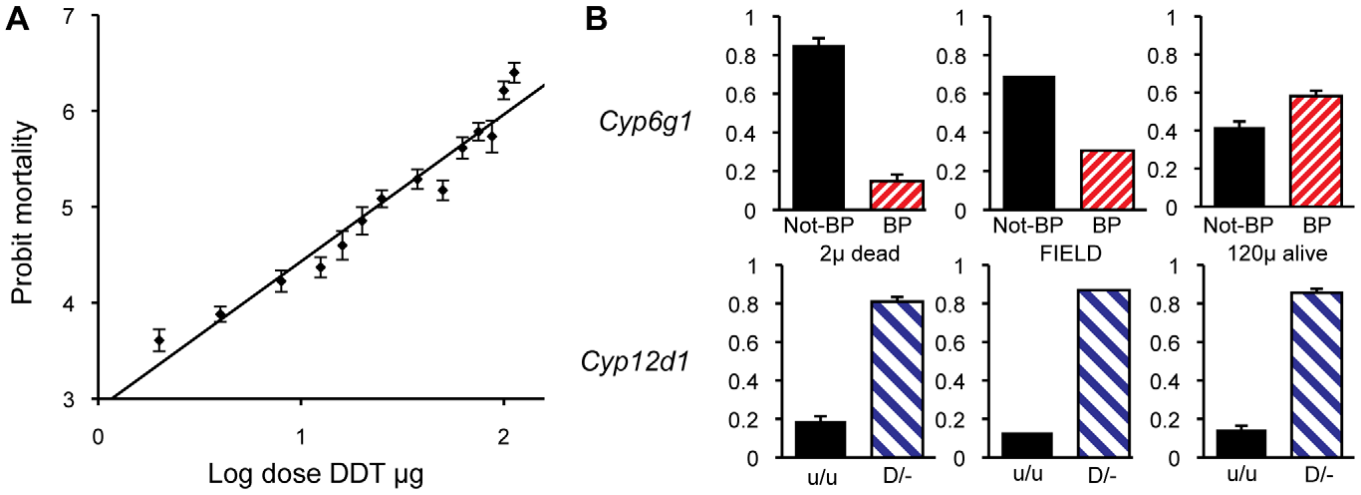
Population analysis of DDT resistance. A DDT association study was conducted on 7500 females derived from a single population. (A) A dosage mortality analysis was undertaken to a identify dose that would kill the 5% of individual that were most susceptible to DDT and the dose that would kill all except the 5% of individuals that were most resistant to DDT. (B) The top three graphs show the allele frequencies of *Cyp6g1-[BP]* among the individuals that died on 2 ug of DDT (frequency of 0.15), among an unscreened field population sample (frequency of 0.31), and among the survivors of 120 ug of DDT (frequency of 0.58), respectively. Thus there is a strong association between DDT resistance and *Cyp6g1*-[*BP*]. The bottom three graphs show the frequency of a *Cyp12d1* copy number variant among the same three groups (*Cyp12d1* D/− = 0.81, 0.87 and 0.86) showing that it is not associated with field resistance. Note that flies carrying the *Cyp12d1* duplication are denoted as D/−, whereas flies homozygous for a single copy are denoted as u/u. Error bars represent standard errors of the mean of biological replicates.

The population for our association study consisted of 7500 non-virgin females that derived from 750 females caught in the field two generations earlier. A subset of our population was used to determine a dosage mortality curve that allowed an estimation of the population LC_5_ and LC_95_ values (i.e. the most susceptible 5% and most resistance 5% of the population to DDT exposure). The mortality of flies on a probit scale was close to linear to the log of the dose of DDT indicating that the underlying phenotype approximates a normal distribution (Figure 6A). Based on this we chose 2 µg/scintillation vial ( = 0.05 µg/cm^2^) of DDT to be the exposure that would only kill the individuals from the susceptible tail of the distribution and 120 µg/scintillation vial ( = 3.2 µg/cm^2^) to be the exposure to kill all but the most resistant individuals. Four replicates of 500 flies were then exposed to the lower dose of DDT and five replicates of 500 were exposed to the high dose of DDT. Nearly exactly 95% of the flies died (all but 124/2500) on the 120 µg treatment and close to 7% of flies died on the 2 µg treatment (133/2000). All the flies that survived the high dose and all those that died on the low dose, were genotyped for *Cyp6g1* allele status. These were compared to 86 field caught males and to random samples of flies that lived on the low dose or died on the high dose. The *BP* allele is greatly enriched above field frequency among survivors of the high dose but was depleted among those that died at the low dose (Figure 6). Thus the association study confirmed the inbred and isochromosomal line analysis. Trend tests give a very significant association between *BP* and survival at LC_95_ (Cochrane Armitage test, chi square 42, p<<0.0001; [37]). In fact flies homozygous for the *BP* allele are twelve times more likely to survive than non *BP* homozygotes.

For comparison we also genotyped the same flies for a CNV at another candidate DDT resistance locus, *Cyp12d1*, which is on chromosome 2 approximately 1 Mb away from *Cyp6g1*. The assay we used bridged the breakpoints of the distal and proximal copies of this locus, which is thus not a codominant assay and therefore individuals either heterozygous or homozygous for the duplication were not discriminated (Flybase release FB2010_01). Therefore a 2×2 test comparing the presence and absence of the *Cyp12d1* duplication in resistant and susceptible flies was used which showed there is no correlation with the duplication of this locus and resistance or susceptibility (Fishers exact test P = 0.60, Figure 6).

These data can also be used to quantify the contribution of *Cyp6g1* alleles to DDT resistance. Table 1 shows the survivorship of individuals partitioned by *Cyp6g1-[BP]* genotype. Heterozygotes are less than midway between the two homozygotes indicating this allele is slightly recessive at this dose (Figure S4). To calculate the contribution of this allele in the population, this recessiveness (k = −0.63), the survivorship difference between the two homozygotes (2a =  0.18), and the population frequency of *Cyp6g1-[BP]* (p = 0.28), need to be taken into account. Thus the average allelic effect of *Cyp6g1-[BP]* is to increase survivorship of a genotype by 6.5% ([38] equation 4.10b). The additive genetic variance attributable to this allele is 0.0017 ([38] equation 4.12a) and therefore the heritability on the observed scale, at an LC_95_ dose, attributable to this allele, is 3.7%. For a threshold trait such as this, we are observing binary phenotypes; alive or dead at a particular dose. However, as illustrated by the dose response relationship in Figure 6A, we can assume that there is a normal distribution underlying the DDT resistance trait across doses. Thus the heritability on the observed scale can be converted to an estimate of the heritability on the underlying scale and that indicates that approximately 16.5% of the ‘liability’ to survive is explained by the *Cyp6g1-[BP]* allele ([38] equation 25.8b). We have not calculated the variance of all genetic factors influencing DDT resistance, and thus do not know the heritability of the trait as a whole, but whatever that heritability is (it has to be between 0.16 and 1), the *Cyp6g1-[BP]* allele makes a major contribution to phenotypic variation in this population.

**Table 1:** Survivorship/mortality of *Cyp6g1-BP* genotypes on 120 µg of DDT.

	Not-BP/Not-BP	Not-BP/BP	BP/BP
Total Alive	27	52	45
Sample from Dead	72	51	10
Total Dead (estimated)	1286	911	179

## Discussion

### An allelic succession at the *Cyp6g1* locus increases DDT resistance

Recently, others have identified CNV at the *Cyp6g1* locus using genome-wide tiling arrays [29], [30]. In the study of Emerson *et al.* the resolution of CNV boundaries is such that repeats containing a fusion of partial *Cyp6g1* and *Cyp6g2* genes were identified. Not only has the present work determined that the CNV represents at least two full-length copies of *Cyp6g1*, it has also established the way in which this locus has evolved. Furthermore, we have shown how this contributes to increasing DDT resistance using two separate approaches. Firstly we showed that the LC_50_ to DDT increases with the allelic progression in a set of 19 isochromosomal and 3 inbred lines (*M*<< *AA*< *BA* << *BP*). Secondly we performed an association study that clearly demonstrates that the *BP* allele makes a major contribution to the phenotypic variance in a single population where it is at high frequency.

### The allelic succession is adaptive

*D. melanogaster* is not a pest and is generally not targeted by insecticide application. Could it be that variation we detect with our DDT assays is non-adaptive? As discussed in more detail below we present historical and geographic surveys of *Cyp6g1* allelic variation that clearly demonstrate that at least two of the steps have been adaptive (the world-wide spread of the *Accord* bearing alleles and the spread of the *BP* alleles within Australia). Further support for adaptive change at this locus could come in the form of patterns in the patterns of polymorphism and fixation that support selective sweep models. In fact others have shown evidence for a selective sweep at the *Cyp6g1* locus [17], [18], although they have not shown that there have been recurrent sweeps at this locus. The data presented here shows that an adaptive walk has occurred at the *Cyp6g1 locus*. Although not a classic adaptive walk, where evolution is conceived in coding sequence space [39], the allelic succession that we describe is explained as a sequential process where each new allele is derived directly from that preceding it. In the following we discuss each proposed step in this allelic succession.

### The first step: insertion of partial *Accord* element

The first step of the walk would seem to have been the insertion of the *Accord* LTR into the *Cyp6g1* promoter. As we did not detect the *A* allele (single copy *Cyp6g1* with *Accord* LTR) in our sample, and thus cannot determine its phenotypic effect, the identification of the AA allele, in which both *Cyp6g1* and the *Accord* LTR are duplicated, indicates that this insertion most likely occurred at or before the duplication event. A question that arises from the failure to detect the single copy *A* allele, is deciding which molecular variant, the partial *Accord* TE insertion or the CNV, was the first target of natural selection. A recent study using transgenic reporter genes showed that the *Accord* LTR acts as an enhancer increasing transcription in tissues consistent with the *Cyp6g1* changes observed in ‘*Accord* flies’ [15]. This suggests the *Accord* insertion itself could cause the up-regulation and be the target of selection.

### The second step: gene duplication

We propose that the second step was the duplication event producing two copies of *Cyp6g1*. Sequence analysis of cDNA from RK146 (not shown) indicates two full-length copies of *Cyp6g1* are transcribed in adult flies, indicating that the duplication acts to increase transcriptional output. It is possible that the *Accord* insertion and the duplication happened from the one complex event. Thus a minimum of one selective sweep is required to explain the rapid change in frequency of the *AA* alleles. Alternatively it is possible that there have been two adaptive steps, one that is the *Accord* insertion and the other the generation of the CNV. In that scenario the *A* allele may never have reached high frequencies before being replaced by the *AA* allele. Whichever the case, the net result is a 7–10 fold increase in resistance phenotype in comparison to the ancestral *M* allele (Figure 4).

### The third step: *HMS-Beagle* insertion

The third step in the walk involved the insertion of the *HMS-Beagle* insertion into the *Accord* LTR that lies proximal to *Cyp6g1-a*. From the DDT resistance data it is hard to determine whether the *AA*→*BA* step is adaptive, as the increase in DDT resistance phenotype is only significant for males in our current data set. Our population surveys confirm previous results, which suggest that the *Accord* alleles (which we now know as *A*, *AA* and *BA* alleles) were either absent or at low frequencies pre-DDT. They also show that both *AA* and *BA* alleles began to spread at the same time, and have now spread globally. Among the lines carrying the *BA* allele is Hikone-R, which was the DDT resistant strain that was initially used to map *Cyp6g1*-based resistance [21], [24]. Hikone-R was collected in Japan in 1952 and although the *AA* and *BA* alleles may have existed for some time before the 1950's, historic fly collections show they have only reached high frequencies recently (Figure 3). These results concur with previously reported skews in the polymorphism frequency spectrum around *Cyp6g1*, which suggests recent strong positive selection [17], [40].

### The fourth step: partial *P*-element insertion

The fourth step in the walk is the insertion of a partial *P*-element into the *Accord* LTR that lies proximal to *Cyp6g1-b*. Since all flies that carry a *P*-element insertion also contain an *HMS-Beagle* element upstream of *Cyp6g1-a* we infer the insertion would have occurred in a *Cyp6g1-[BA]* background. This results in a significant increase in DDT resistance phenotype over *AA* and *BA* alleles with *BP* males 6 and 3 fold more resistant respectively. *BP* females are 8 and 5 fold more resistant than their *AA* and *BA* counterparts. Furthermore the association study shows a highly significant association between the *BP* allele and DDT resistance.

Curiously, the robust association between the *BP* allele and the DDT resistance phenotype is not reflected in our transcriptional analysis. This may be because the *P-*element insertion may simply be a marker in linkage disequilibrium with the causal variant – which could be an amino acid change in an uncharacterised copy of *Cyp6g1*. Another possibility is that the *BP* allele gives resistance by altering the transcript abundance in tissues that have not been assayed here. The possibility that there is tissue specific variation in transcript levels is illustrated by the observed differences in expression between Malpighian tubules and midgut. Thus it is possible, for instance, that *Cyp6g1* transcription is higher in the head, fat body or reproductive tissues in *BP* lines.

Regardless of the exact details of the molecular mechanism of resistance we have no doubt that the fourth step is adaptive, as analysis of eight Australian populations suggests the *Cyp6g1-[BP]* variant has recently and rapidly increased to be the most frequent allele in Australia. Thus Australian flies are very different from other parts of the world where *BP* alleles were recorded in only 10 out of 683 lines [17]. The lack of *BP* alleles in fly lines established from Papua New Guinea and Australia in the 1980s (Figure 4A) supports this selection model as do reports that the *P-*element transposable element itself was only detected in Australia in the late 1970's [41].

Neither drift nor population bottlenecks can satisfactorily account for the high frequency of *BP* alleles in Australia. We conducted three independent surveys of contemporary Australian populations (our original survey, a survey of east Australian clinal samples and the association study collection) sampling from multiple locations spanning the east coast of Australia. Thus a local bottleneck (i.e. from a single collection site) could not explain the data. Similarly *D. melanogaster* populations from the east coast of Australia exhibit extensive gene flow and share the same diversity as non-African populations [35], [42], indicating populations are not isolated. There has also been enough time since their introduction to Australia for the establishment of strong latitudinal clines that parallel those found in other parts of the globe [43]–[45]. Furthermore the flies used here to survey allele frequency across the east coast of Australia have been previously characterized for many other loci and are consistent with other Australian surveys, ruling out the possibility that our samples are somehow biased, non-representative or corrupted[43]. Finally if the *P* element did not enter Australia until the 1970's [41] then the *BP* alleles of *Cyp6g1* must have entered into established populations. There is no way that genetic drift could explain the frequencies of *BP* alleles, rather the *BP* alleles must have spread through these populations with positive selection. This raises the interesting proposition that the *BP* alleles may increase in frequency in other parts of the world in the future.

It is worth noting that DDT has been banned from use in most of the world including Australia since the 1980s [46] and yet we are postulating that the *BP* allele has risen to high frequency in Australian populations since then. Notwithstanding the possibility that DDT still persists in the environment, we also note that it is well established that *Cyp6g1* upregulation provides resistance to a number of insecticides and other chemicals [13], [24]. Thus DDT resistance may be considered as a phenotypic marker of this allelic variation rather than the actual selective agent.

### Further steps: more CNV and TE sequence deletion

Our population surveys also identified two different *Cyp6g1-[BP*Δ*]* alleles, formed by imprecise excision of the *P*-element insertion. These alleles are at low frequency, and we have not characterised their contribution to DDT resistance. Their formation questions the stability of the locus structure that we have defined. Over evolutionary time we would expect this structure to be simplified. For instance if the *BP*Δ alleles have the same phenotype as their *BP* parent, it may indicate that only discrete functional DNA sequences need be preserved, with the rest free to be deleted. Opposing this simplification is the instability introduced by the gene duplications, which may increase the rate of copy number variation by molecular slippage. We have shown that in the RK146 strain there are at least two full-length copies of *Cyp6g1*, but in light of the above it is possible that even more copies exist in other strains.

### Precedence for allelic succession

Allelic succession, the process whereby different adaptive alleles are substituted sequentially, has also been characterised in several studies of insecticides resistance. In *Culex pipiens* mosquitoes, alleles at the *Ester* ‘super-locus’ (so called because some alleles contain CNV of more than one gene) confer organophosphate (OP) resistance. *Ester^1^* and *Ester^4^* both result in the overproduction of an insecticide metabolising esterase [47]. *Ester^1^* was first detected in 1972, while *Ester^4^* appeared over a decade later. Despite a moderately lower level of OP resistance *Ester^4^* has replaced *Ester^1^* due to a lower overall fitness cost [48]. In a second *Culex* example, the resistance allele of the target of OP insecticides, *Ace1^R^*, also confers a fitness cost, but this cost seems to have been reduced through gene duplication by the creation of a permanent heterozygous allele, consisting of a copy each of the susceptible and resistant alleles [49]. Allelic succession in both these cases appears to be driven by selection removing a fitness cost introduced with the preceding resistance allele.

Two previous studies have associated fitness costs with *Cyp6g1* upregulation. A study in which males were selected for reduced competitive mating success indicated that *Cyp6g1* was significantly upregulated [50]. In contrast, females seem to carry no cost for a range of fitness traits [51]. It would be worthwhile revisiting these earlier experiments in light of the complex variation we have shown to exist for *Cyp6g1*. However it is not necessary to invoke fitness costs, as the data shown in Figure 4 suggests that the allelic succession occurring at *Cyp6g1* is driven by selection for ever-greater resistance.

### Concluding remarks

*Cyp6g1* has become a highly cited example of adaptive evolution [52]–[54]. *Cyp6g1* resistance alleles are not only selected, in parallel, in sibling species, we show that they have also been repeatedly selected, in series, in *D. melanogaster*. These results are pertinent to a long-standing evolutionary debate concerning the number of steps that have to occur to move a species to a new adaptive optimum [6], [55]. Support for a model requiring only a moderate number of steps includes mapping experiments, where the number of loci that contribute to a given adaptive trait are calculated, and their relative phenotypic effects apportioned. However, as recently described by the careful dissection of a morphological trait that differs between species, mapping experiments may hide the number of steps that have occurred at a single locus over evolutionary time [56]. Here we have described at least four steps at a single locus that have occurred within 70 years. The intense selection of insecticides has provided the opportunity to see the adaptive process at a resolution invisible in many other examples of adaptation.

## Materials and Methods

### Fly stocks

The stocks used for the DDT toxicology experiment were made isochromosomal for the II chromosome by backcrossing to *Prl/CyO* flies (Bloomington Stock 3079). For stock list see Table S2.

### Polytene chromosomes

Probes were made using the PCR DIG Probe Synthesis kit of Roche Boehringer Mannheim (version# 2003). The primers: 5′-CAGCCTAGAGAATCCCAACG-3′ and 5′GCCATGGCCACTATGTTCTT-3′ were used to amplify exon 3 and exon 4 from a *Cyp6g1* subclone. The chromosomes were prepared following the method of Phillips *et al* [57].

### Long PCR

Roche Expand High Fidelity PCR system was used to generate all PCR products greater than 2.5 kb following the manufacturers protocol except with the following cycling parameters: 94°C 2 mins, 10 cycles of 94°C 15s, 62°C* 30s, 68°C 10 mins, 30 cycles of 94°C 15s, 56°C 30s, 68°C 10^#^ mins, and a final 60 min extension at 68°C. * A touchdown cycle, with the annealing temperature decreasing by 0.5°C per cycle. ^#^ Extension time increases 10 s per cycle. Primers used (refer Figure 2) listed 5′-3′: **A** CGTCTTAGAAAGAAACAGGAAACTG, B ACATTTGGGAGATGCCTTTG, C ATTAAACACAACCGGCTTTCTCG, D GTCTCACCACCCAGGAAAGA, **E** CTTTTTGTGTGCTATGGTTTAGTTAG, **F** GGGTGCAACAGAGTTTCAGGTA, **G** TTTCAGCCAGTTGGACATTG. PCR products were gel purified and cloned using the TOPO XL PCR Cloning Kit (Invitrogen) following manufacturers instructions.

### Inverse PCR

125 ng of RK146 genomic DNA was digested with EcoR1 in a total volume of 100 uL, then 5 uL of the digest was diluted to 100 uL in a ligation reaction mix, left overnight at 14°C. This allowed linear EcoR1 fragments to circularise. PCR's using primers 5′-GATCCGCGGCTGAAGGACGA-3′ and 5′-TGCGGCGACCACCACAAAGA-3′ were conducted with the 30 cycles of: 94°C for 30 s, 62°C for 30 s and 68°C for 2minutes. A nested PCR was then performed using a new reverse primer (5′-TGCCAGTGCCCTCAGCATTATCTTATC-3′) and the original forward primer (5′-GATCCGCGGCTGAAGGACGA-3′). The product was cloned into pGEM-T easy and sequenced.

### Allele scoring

DNA was prepared from single flies. Diagnostic assays to detect TE insertions and the *Cyp6g1* gene duplication used standard PCR conditions with the following cycling parameters: 94°C 2 mins, 30 cycles of 94°C 15 s, *°C 30 s, 72°C # mins. Reactions used the following primers (refer Figure 2) listed 5′-3′: **H** GAAAGCCGGTTGTGTTTAATTAT, I CTTTTTGTGTGCTATGGTTTAGTTAG, **J** CGAGTACGAGAGCGTGGAG, **K** ATTAAACACAACCGGCTTTCTCG, **L** TGCGATCATCTGCACTTCTC. Annealing temperatures, *, and extension times, #, for each primer pair: HI 57°C 2 mins, JK 56°C 1 min, LI 58°C 45 secs.

### DDT resistance assays

4 day old non-virgin male and female flies were treated separately. DDT was coated on the inside of glass scintillation vials by applying 200 µl of acetone containing varying concentrations of DDT and rolling the vial until the acetone had evaporated. 20 flies per vial were used with the vials plugged with cotton soaked in 5% sucrose. Mortality was scored after 24 h. LC_50_ estimation was performed using PriProbit[58], using five concentrations and three replicates per concentration.

### Analysis of gene expression

Midguts and Tubules were dissected separately from 4 day old adult males, 5 strains per genotype, and pooled in groups of 6–10 tissues, for 3 biological replicates per strain. mRNA was extracted in 200 ul Trizol and 60 ul chloroform. After being pelleted all the extracted RNA from each sample was used in cDNA synthesis, and cDNA was reverse transcribed and quantified according to standard procedures. For quantitative PCR (qPCR), samples were split and amplified with *Cyp6g1* primers, using *Rpl11* as a reference gene.

### DDT association study

750 isofemale lines were established from field caught females. At the F2, 10 4–8 day non-virgin females were collected from each line, in essence recapitulating the extant genetic variation of this population. Our experimental design involved comparing the two tails of the DDT resistance phenotype distribution. To this end a subset of the F2 flies were used to determine a dosage mortality curve for the population and allow estimation of the population LC_5_ and LC_95_ values (2 µg and 190 µg respectively). The large 95% confidence interval for the LC_95_ estimate suggested a dose of 190 µg would be inaccurate, so instead we used a dose of 120 µg, a dose slightly higher than the highest dose used in the DMC assay (112.5 µg) which had ∼92% mortality. These doses were scaled, by internal surface area, to allow exposure of 500 individuals in 2 L Schott bottles, which were stoppered by cotton wool wrapped around a 10 ml disposable pipette. After exposure for 24 hours a vacuum pump was attached to the pipette to remove the flies into separate dead and alive cohorts. For the LC_5_, all dead flies (133 in total) and an equivalent number of surviving flies were assayed. For the LC_95_ all surviving flies (124) and an equivalent number of dead flies were assayed. Flies were assayed using the HI primer pair to detect *Accord/P*-element status as described above. Determination of the *Cyp12d1* duplication genotype status (u/u or D/−) of these flies utilised a four primer PCR reaction using the same general PCR conditions described above. Primers used were; Rout TCCTAAGAATTCCCACCATCAC, Rin GGTCCATCATCCCTACCATTT, *Fout GGCCATTACGTTCCCCTTC and Fin GGTCTCGGAAAATGAGCAAC. The Rin/Fout and Rout/Fin primer pairs amplify products from both single copy and duplicated* Cyp12d1 *loci 767 bp 933 bp in length respectively. The pair Rout/Fout is specific to the presence of the gene duplication, and gives a product of 389 bp*.

## Supporting Information

Figure S1 CNV at *Cyp6g1* is limited to one cytological band. In situ hybridisation of a DIG labelled *Cyp6g1* probe to a polytene chromosome spread of the RK146 strain. The probe was created from exon 3, intron 3 and part of exon 4 of *Cyp6g1*. The inset magnifies the hybridisation, on chromosome arm 2R, and indicates the presence of the *Cyp6g1* gene duplication within the one cytological band.
(1.43 MB PDF)

Figure S2 Southern Blot analysis of *Cyp6g1*. A Southern blot of RK146 genomic DNA probed with a PCR product derived from exon 3 and exon 4 of *Cyp6g1* (flanked with primers cgagtacgagagcgtggag and acatttgggagatgcctttg). Note that the repeat structure of the locus is indicated by the probe hybridizing to bands of the same size in DNA cut with different enzymes. A 2.7 kb band, seen in SacI, HpaI and EcoRV digests corresponds to the size of the repeat consisting of partial *Cyp6g1/Cyp6g2* sequences (Figure 1). The large 11.4 kb band in AflII, EcoR1 and PstI fragments reflects the distance between the two full length sequences. Note that the ∼8 kb band in AflII, EcoR1 and PstI suggest that there may be a third copy of this sequence. B. The probe binding sites (thick black blocks below the locus model) are shown with respect to the restriction enzyme map and our locus model (upper right). Approximate migration of the molecular weight markers are shown on the left.
(1.27 MB PDF)

Figure S3 The sequence composition of the BP delta lines. The top sequence is a fragment of the Accord insertion (from position 297 of AY131284). The second sequence shows the partial P element insertion (in green) previously described within the Accord sequence (BP lines; [31]). The duplicated 8 bp target sites are shown in purple. Pder1 and Pder2 are sequences from two BP- delta lines (N89 and Mb59) collected in Australia. The 31 bp terminal repeat of the canonical P-element is shown at the bottom and is annotated (with cyan, italics and underlining) to reflect the sequences observed in the P-derived alleles. The sequence marked in cyan in Pder1 and Pder2 is the reverse complement of the sequence marked cyan in the terminal repeat.
(0.03 MB PDF)

Figure S4 Quantifying *Cyp6g1* BP's contribution to high level resistance. The percentage survival at 120 ug for each genotype is plotted allowing for the calculation of the allelic affect for *Cyp6g1*, a = 0.09. The survivorship of the *Cyp6g1* BP heterozygote is less than that expected (indicated by the point in red), so is recessive (k = −0.63). This leads to a modified affect of α = 0.07. This allows calculation of the narrow sense heritability for Cyp6g1 BP of 3.7% on the observed scale, or 16.5% on the underlying liability scale. Error bars represent standard error of mean of 5 biological replicates of 500 flies each.
(0.32 MB PDF)

Table S1 Differences between two *Cyp6g1* copies in the RK146 strain. Site refers to co-ordinates in gDNA relative to predicted transcription start site.
(0.22 MB PDF)

Table S2 Fly stocks used in this study. Shows the name, origin, date of collection, supplier, degree of inbreeding and *Cyp6g1* allele type of the fly lines used in this study.
(0.05 MB XLS)

Table S3 LD50 of inbred and isochromosomal lines.
(0.04 MB PDF)
