# Supplementary figures and images for: Copy Number Variation and Transposable Elements Feature in Recent, Ongoing Adaptation at the Cyp6g1 Locus
Source: PLoS Genet. 2010 Jun 24;6(6):e1000998. doi: 10.1371/journal.pgen.1000998 (PMC2891717; doi:10.1371/journal.pgen.1000998)

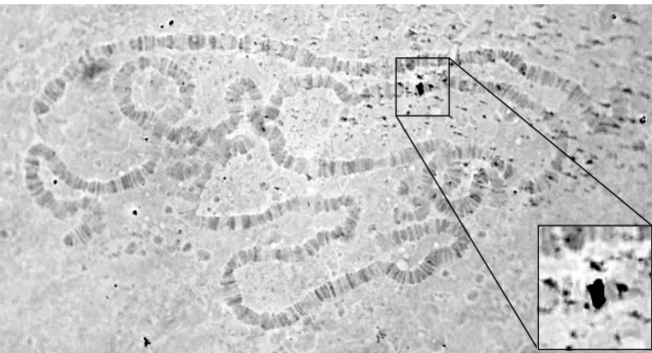

Supplement: Figure S1 — CNV at Cyp6g1 is limited to one cytological band. In situ hybridisation of a DIG labelled Cyp6g1 probe to a polytene chromosome spread of the RK146 strain. The probe was created from exon 3, intron 3 and part of exon 4 of Cyp6g1. The inset magnifies the hybridisation, on chromosome arm 2R, and indicates the presence of the Cyp6g1 gene duplication within the one cytological band. (1.43 MB PDF) [file pgen.1000998.s001.pdf]

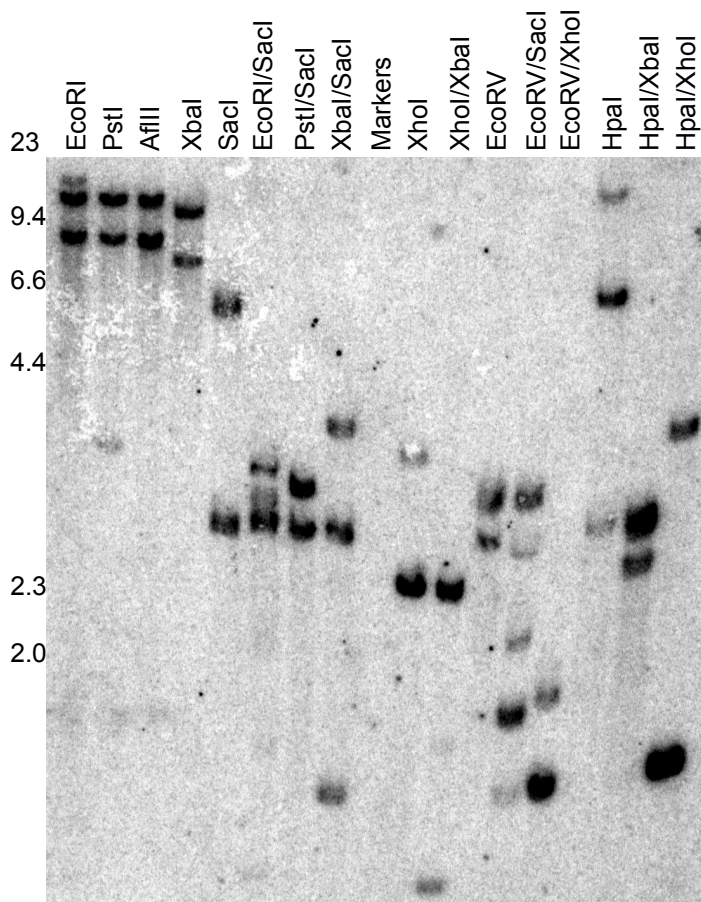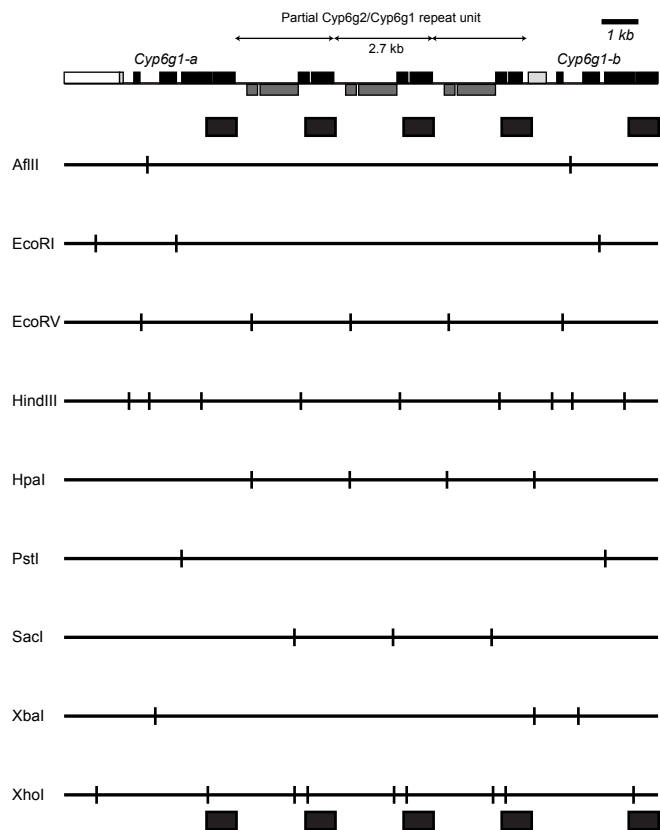

Supplement: Figure S2 — Southern Blot analysis of Cyp6g1. A Southern blot of RK146 genomic DNA probed with a PCR product derived from exon 3 and exon 4 of Cyp6g1 (flanked with primers cgagtacgagagcgtggag and acatttgggagatgcctttg). Note that the repeat structure of the locus is indicated by the probe hybridizing to bands of the same size in DNA cut with different enzymes. A 2.7 kb band, seen in SacI, HpaI and EcoRV digests corresponds to the size of the repeat consisting of partial Cyp6g1/Cyp6g2 sequences (Figure 1). The large 11.4 kb band in AflII, EcoR1 and PstI fragments reflects the distance between the two full length sequences. Note that the ∼8 kb band in AflII, EcoR1 and PstI suggest that there may be a third copy of this sequence. B. The probe binding sites (thick black blocks below the locus model) are shown with respect to the restriction enzyme map and our locus model (upper right). Approximate migration of the molecular weight markers are shown on the left. (1.27 MB PDF) [file pgen.1000998.s002.pdf]

% Genotype alive at 120 $\mu$ g DDT

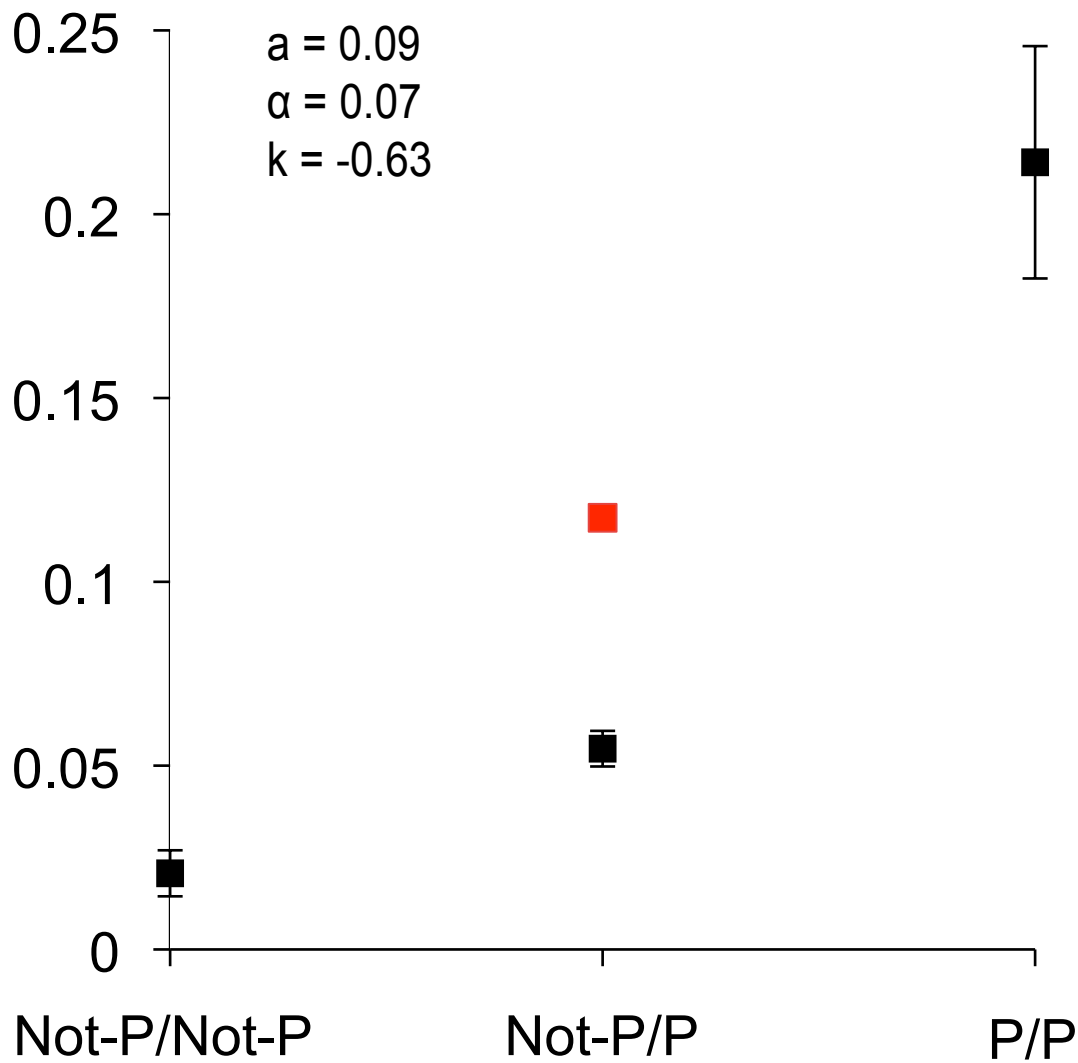

Genotype

Supplement: Figure S4 — Quantifying Cyp6g1 BP's contribution to high level resistance. The percentage survival at 120 ug for each genotype is plotted allowing for the calculation of the allelic affect for Cyp6g1, a = 0.09. The survivorship of the Cyp6g1 BP heterozygote is less than that expected (indicated by the point in red), so is recessive (k = −0.63). This leads to a modified affect of α = 0.07. This allows calculation of the narrow sense heritability for Cyp6g1 BP of 3.7% on the observed scale, or 16.5% on the underlying liability scale. Error bars represent standard error of mean of 5 biological replicates of 500 flies each. (0.32 MB PDF) [file pgen.1000998.s004.pdf]
