# Supplementary material for: Copy Number Variation and Transposable Elements Feature in Recent, Ongoing Adaptation at the Cyp6g1 Locus
Source: PLoS Genet. 2010 Jun 24;6(6):e1000998. doi: 10.1371/journal.pgen.1000998 (PMC2891717; doi:10.1371/journal.pgen.1000998)
Supplement: Figure S3 — The sequence composition of the BP delta lines. The top sequence is a fragment of the Accord insertion (from position 297 of AY131284). The second sequence shows the partial P element insertion (in green) previously described within the Accord sequence (BP lines; [31]). The duplicated 8 bp target sites are shown in purple. Pder1 and Pder2 are sequences from two BP- delta lines (N89 and Mb59) collected in Australia. The 31 bp terminal repeat of the canonical P-element is shown at the bottom and is annotated (with cyan, italics and underlining) to reflect the sequences observed in the P-derived alleles. The sequence marked in cyan in Pder1 and Pder2 is the reverse complement of the sequence marked cyan in the terminal repeat. (0.03 MB PDF) [file pgen.1000998.s003.pdf]

Accord_LTR TTTTGATCCCGTTAGTTCTAGATAGCGA**GTCTAGAC**--GAGGACGTCGCGATTACGCAGTGTGTATGTGTGTGTGT

BP lines TTTTGATCCCGTTAGTTCTAGATAGCGA**GTCTAGAC**←31bp_ITR____0.9kb_partial_P___31bp_ITR→**GTCTAGAC**GAGGACGTCGCGATTACGCAGTGTGTATGTGTGTGTGT

Pder1_N89 TTTTGATCCCGTTAGTTCTAGATAGCGA**GTCTAGAC***CATGATGAAATAACATA*TGAAATAACATGTTATTTCATCATG**GTCTAGAC**GAGGACGTCGCGATTACGCAGTGTGTATGTGTGTGTGT

Pder2_Mb59ii TTTTGATCCCGTTAGTTCTAGATAGCGA**GTCTAGAC***CATGATGAAATAACATA*TG----------TTATTTCATCATG------------ACGTCGCGATTACGCAGTGTGTATGTGTGTGTGT

The canonical 31bp terminal repeat of a P-element: *CATGATGAAATAACATA*AGGTGGTCCCGTCG
