# Supplementary material for: Copy Number Variation and Transposable Elements Feature in Recent, Ongoing Adaptation at the Cyp6g1 Locus
Source: PLoS Genet. 2010 Jun 24;6(6):e1000998. doi: 10.1371/journal.pgen.1000998 (PMC2891717; doi:10.1371/journal.pgen.1000998)
Supplement: Table S1 — Differences between two Cyp6g1 copies in the RK146 strain. Site refers to co-ordinates in gDNA relative to predicted transcription start site. (0.22 MB PDF) [file pgen.1000998.s005.pdf]

**Site**	1277	1283	1344	1392	1479	1827	1911
**Polymorphism**	**R**	**W**	**M**	**Y**	**Y**	**Y**	**R**
Cyp6g1 a	A	A	C	C	T	C	A
Cyp6g1 b	G	T	A	T	C	T	G
