# Supplementary material for: Copy Number Variation and Transposable Elements Feature in Recent, Ongoing Adaptation at the Cyp6g1 Locus
Source: PLoS Genet. 2010 Jun 24;6(6):e1000998. doi: 10.1371/journal.pgen.1000998 (PMC2891717; doi:10.1371/journal.pgen.1000998)
Supplement: Table S3 — LD50 of inbred and isochromosomal lines. (0.04 MB PDF) [file pgen.1000998.s007.pdf]

		Male		Female	
		LD50	95% CI	LD50	95% CI
M					
	Celera	0.48	0.38-0.51	0.64	0.28-1.5
	BG4	1.4	0.93-1.6	3.6	3.1-4.2
	Amherst 3	0.46	0.30-1.3	0.99	0.73-1.2
	ICC 37	1.8	0.8-1.7	0.46	0.2-1.3
	MW 46-2	0.81	0.70-1.0	0.53	0.50-0.63
AA					
	C7	11.3	7.73-22.8	11.2	9.8-12.9
	DJ33i	3.6	0.96-5.74	10.9	9.2-12.5
	RB117	7.4	5.81-10.42	14.8	10.5-27.3
	RK24	5.4	4.50-6.42	13.5	10.4-19.2
	ICJ 84	4	1.5-7.3	6	4.3-10.5
	TR2	9.1	7.20-12.33	19.6	15.8-25.3
BA					
	RK146	2.9	2.3-3.4	8.1	2.5-13.4
	Hikone-R	15.2	12.5-18.8	24.3	20.2-29.4
	ICJ 68	16	13.7-19.2	22	17.9-26.5
	ICC 70	12	10.9-18.1	16	14.6-21.9
	MV8ii	16.4	11.6-31.0	22.6	18.2-27.9
BP					
	K87i	66.0	48.2-86.2	241.0	196.1-300.6
	CT49B	28.0	23.4-33.4	63.9	54.7-74.0
	DJ21	33.0	27.0-39.6	86.7	52.9-109.7
	N12	42.8	33.9-52.7	93.2	50.9-104.0
	ICC 18biv	35	27.9-42.3	50	43.6-74.3
	A97	28.9	14.2-40.0	67.2	52.6-80.4
